# Companion Plants of Tea: From Ancient to Terrace to Forest

**DOI:** 10.3390/plants12173061

**Published:** 2023-08-25

**Authors:** Huan Wu, Xiaofeng Long, Yanfei Geng

**Affiliations:** College of Tea Science, Guizhou University, Guiyang 550025, China; hwu2023@163.com (H.W.); xflong66662022@163.com (X.L.)

**Keywords:** companion plants, forest tea gardens, ancient literature, sustainable development

## Abstract

China is one of the origins of ancient tea gardens, with a long history of tea culture and tea cultivation. Guizhou Province is an important tea production place in southwest China with rich forest tea resources. The purpose of this study is to obtain historical information on companion plants in historical tea gardens and provide a theoretical basis for the sustainable development of forest tea gardens in Guizhou Province. We conducted a statistical analysis and comparison of plant species among ancient tea gardens, terrace tea gardens, and forest tea gardens from a diachronic perspective, based on 21 ancient tea literature studies, 116 terrace tea garden documents, and 18 sampled plots of forest tea gardens in Guizhou. A total of 24 companion plants species belonging to 16 families and 22 genera were found in ancient tea gardens, 81 species were found in terrace tea gardens belonging to 37 families and 74 genera, and 232 species were found in sample plots of forest tea gardens belonging to 90 families and 178 genera. Companion plants can be divided into three categories. Most of the plant families recorded in the literature also appeared in the forest tea garden we surveyed. In ancient tea gardens, terrace tea gardens, and forest tea gardens, Poaceae, Fabaceae, and Rosaceae were the most dominant families, respectively. The intercropping of tea gardens has been practiced since ancient times. Companion plants in natural forest tea gardens not only provide important insights into intercropping of terrace gardens but also hold significant implications for the conservation of existing forest tea gardens and the sustainable development of tea gardens.

## 1. Introduction

Tea has a long history in China. Tea plants originate from forests. They are one of the characteristic and important tree species of subtropical evergreen broad-leaved forests. In a stable plant community structure, they are found as tall trees or shrubs in the understory of the forest. According to legends, Shennong, an ancient Chinese ruler, tasted various herbs and encountered seventy-two toxic substances in a single day but was healed by tea (神农尝百草，一日遇七十二毒，得荼而解之), which was believed to be the main function of tea in ancient China. In the Ming Dynasty, Shen Yang’s “Dan Qian Record” (the year 1547) states that “tu 荼 is the ancient word for tea 茶. Additionally, Yu Lu’s “The Classic of Tea” (the year 780) from the Tang Dynasty mentioned that tea originated from the Shennong family 茶之为饮，发乎神农氏. According to Gu Ban’s “White Tiger Tongyi” (the year 79) in the Eastern Han Dynasty, the Shennong period was the primary agricultural stage of China’s Urgesellschaft. At present, archaeological discoveries have revealed that the Shennong period began over 10,000 years ago, and it can be inferred that the history of tea might also extend over 10,000 years [1].

Chinese tea culture has a rich and extensive history, dating back to the Pre Qin period, appearing also in the literature of the Jin Dynasty and Han Dynasty, and thriving in the Tang Dynasty and Song Dynasty. “The Classic of Tea” (the year 780) written by Yu Lu marks the formal establishment of Chinese tea culture and also allows the study of Chinese tea culture to enter the academic field of vision [2]. The emergence of the “The Classic of Tea” (the year 780) led to the rise of the tea literary works, such as Tingyun Wen’s “Tea Picking Record” (the year 860), Ran Jiao’s ”Cha Jue” 茶决 (the year 784), Wen Fei’s ”Cha Shu” 茶述 (the years 811–813), etc. During the Song and Yuan Dynasties, the trend of drinking tea flourished day by day, and tea was an indispensable drink in the daily lives of the people from the emperor to the countryside. Moreover, there were stunning verses such as “ Tea is for civilian use, equivalent to rice and salt, and cannot be consumed without it for a day” in Anshi Wang’s “Yi Cha Fa” 议茶法 (the year 1059) of the Song Dynasty, and “those what are indispensable every day are firewood, rice, oil, salt, sauce, vinegar and tea”, recorded in Zimu Wu’s “Dream of Liang Lu” (the year 1274) of the Song Dynasty. In addition, tea-drinking customs such as tea parties, tea competitions, and tea banquets are also quite popular, and this further illustrates the important position of tea at that time [3,4].

The cultivation of tea plants in China also has a long history. Chaosheng Wang [5] inferred that the tea planting by ancient people in China has a history of at least 2000 years through his understanding of the two ancient tea literatures “Huayang Guo Zhi” (the years 348–354) and “Sichuan General Records” (the year 1816). The earliest records of tea tree cultivation techniques in China can be traced back to “Guang Zhi” (the year 270), which was written in the Western Jin Dynasty. There are also numerous pieces of literature from later dynasties that record the cultivation of tea plants. Lin Luo‘s “Cha Jie” 茶解 (the year 1609) of the Ming Dynasty recorded the method of preserving tea seeds to facilitate planting in the coming year. “Dongxi Shi Cha Lu” (the years 1049–1054) and “Da Guan Cha Lun” (the years 1107–1110) of the Song Dynasty recorded “Tea is suitable for the shade of high mountains, but prefers the early morning of the sun”. “Beiyuan Bie Lu” (the year 1196) and “Cha Jie” (the year 1609) of the Song and Ming Dynasty recorded “where was better place for tea plantation, and what were good companion trees to tea”.

During the Ming and early Qing dynasties in China, the Tea culture was a stage of stable and sustainable development, but in the end of Qing Dynasty, it was a stage of abnormal development followed by rapid decline [6]. In the Ming Dynasty, not only did the cultivation of tea plants expand but later Ming treasure voyages spread Tea culture further. Moreover, there was a big improvement in tea cultivation techniques with the emergence of tea seedling transplanting methods and tea garden intercropping practices. In the early Qing Dynasty, the area of tea plantations continued to expand, and asexual propagation methods of tea branch cutting and layering were invented [7,8]. There have also been further improvements in tea garden management, such as tea tree pruning, weeding, and fertilization. From the late Qing Dynasty to the establishment of the People’s Republic of China, Chinese tea farming entered a period of decline [9,10,11,12].

After the establishment of the People’s Republic of China, the tea economy developed gradually. During this period, great efforts were devoted to developing terrace tea gardens, from learning methods to improve terrace tea garden management to increasing investment in labor, funds, fertilizers, and pesticides in terrace tea gardens. As a result, the area of terrace tea gardens was expanded, and the yield of terrace tea gardens was significantly increased nationwide. For example, from 1954 to 1965, the terrace tea garden in Yuhang Tea Experimental Field increased its area by 153.3 hectares, with an average yield of 1200 kg per hectare [13]. According to the data from the China Tea Marketing Association, the total area of China’s terrace tea gardens reached 3.165 million hectares in 2020. Although the development of terrace tea gardens in this way can play a great role in the economy, there are serious problems such as ecosystem instability caused by single planting, ecological environment destruction, pesticide residues, and chemical fertilizer residues [14,15,16,17]. 

In December 2022, Professor Shengji Pei defined a Forest Tea Garden as “it is a tea community of the genus *Camellia* (sect. Thea, including *C. sinensis* var. *sinensis*, *C. sinensis* var. *assamica*, *C. sinensis* var. *dehungensis*, *C. taliensis*, *C. crassicolumna*) of family Theaceae, managed in a traditional way, meeting the green and organic planting standards, with the goal of producing ecological tea and an area of more than 0.5 hm^2^, accompanied by at least one other wild or cultivated woody plant that forms a canopy over 10% and reaches a height of over five meters” [18]. Guizhou Province is an important tea production area in Chinese history, and it was also one of the eight major tea regions in China during the Tang and Song dynasties, known as the “Qianzhong Tea Region” [19]. Records of forest tea in Guizhou could be traced back to Yu Lu’s “The Classic of Tea”. It was written that the taste of “Du Ru Gao Shu Tea” (also known as Wu Chuan Da Shu Tea) was highly enjoyable. Tingjian Huang of the Song Dynasty also praised the Du Ru Gao Shu Tea of Qianyang, which had a bitter taste and helped to relieve the dizziness in his “Jian Cha Fu” (the years 1057–1061). Guizhou Province is rich in wild tea forest resources. According to the survey, Fanjingshan Nature Reserve in Tongren City alone has an area of 13,700 hm^2^ of wild tea forest [20].

The purpose of this study is to conduct a diachronic comparison of the similarities and differences in companion plants among ancient tea gardens, terrace tea gardens, and forest tea gardens by analyzing ancient tea literature and terrace tea garden literature, as well as a sample plot survey. Also, it aims to provide a theoretical basis for the sustainable development of forest tea gardens in Guizhou Province.

## 2. Materials and Methods

### 2.1. Review of Ancient Literature in China before the Late Qing Dynasty

**Figure 1:**
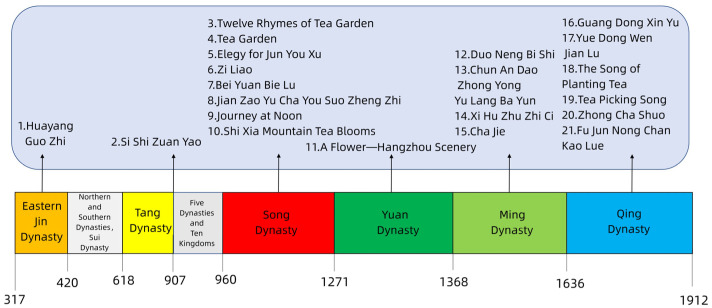
Historical records of major ancient tea literature.

In this study, 21 pieces of ancient tea literature including articles, books, and poetry written before the end of the Qing Dynasty of China were selected using the keywords “ancient”, “tea garden”, “companion plants”, and “intercropping” (Figure 1). The author’s name, literature name, the year of completion, the Chinese name of companion plants, and reference fragments regarding companion plants of ancient tea literature were summarized (Table 1). 

### 2.2. A Survey of Companion Plants in Terrace Tea Gardens after the Establishment of the People’s Republic of China

One hundred and sixteen Chinese documents regarding companion plants in terrace tea gardens were found in the CNKI database (www.cnki.net, accessed on 16 August 2023) using the keywords “tea garden”, “intercropping”, “companion plants”, “ecological benefit”, “terrace tea garden”, and “tea growth”. The types of publications were articles and reviews, and the period of publication was 1957 to 2022. 

### 2.3. Forest Tea Garden Survey

#### 2.3.1. Overview of the Survey Area

**Figure 2:**
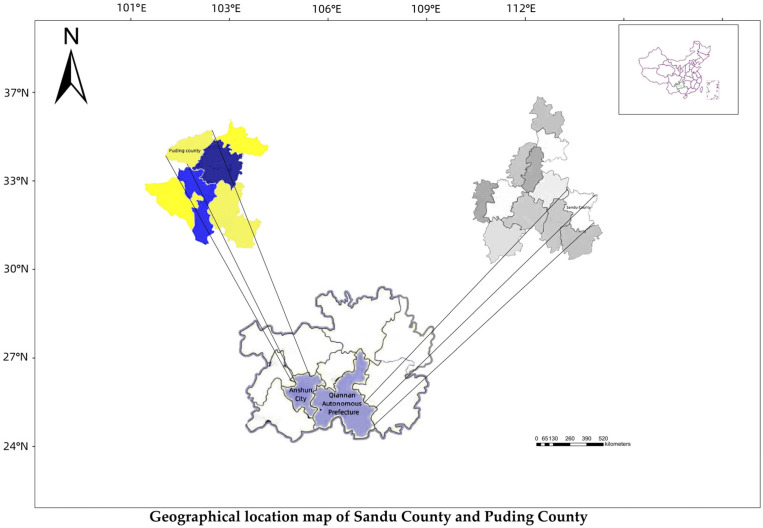
Location of the forest tea gardens in Guizhou Province, China.

(1) Puding County (longitude 105°27′ E to 105°58′ E and latitude 26°26′ N to 26°31′ N) is located northwest of Anshun City in the middle of Guizhou Province (Figure 2). It has a subtropical monsoon humid climate.
(2) Sandu (longitude 107°40′ E to 108°14′ N and latitude 25°30′ N to 26°10′ N), located southeast of Qiannan Buyi and Miao Autonomous Prefecture in Guizhou Province, is the only autonomous county of Shui nationality in China (Figure 2). It is located in the hinterland of “Moon Mountain and Leigong Mountain”. It has a temperate subtropical monsoon climate.

#### 2.3.2. Sample-Plot Survey

**Figure 3:**
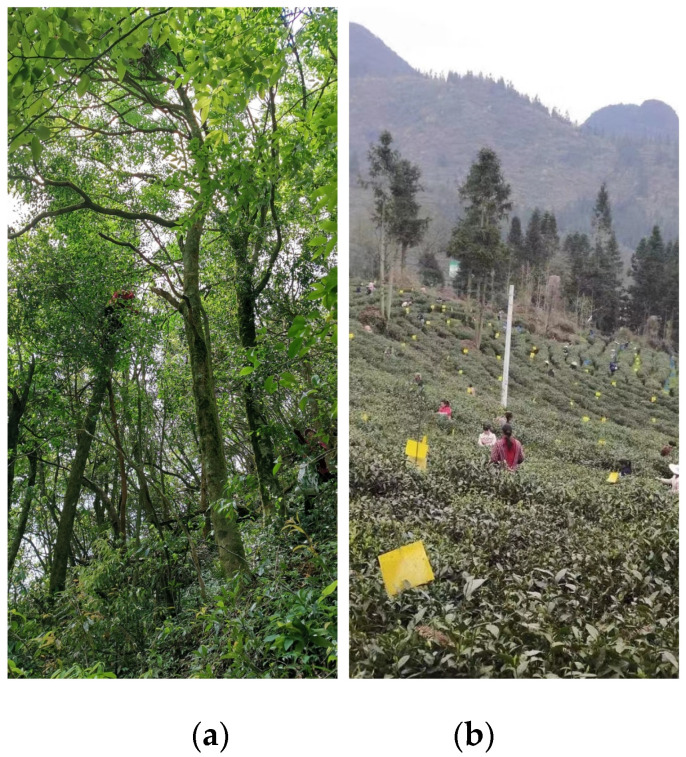
The comparison of forest tea gardens and terrace tea gardens in Guizhou, China. (**a**) Forest tea garden. (**b**) Terrace garden.

Eighteen sample plots were chosen in the forest tea gardens located in Sandu County and Puding County, Guizhou Province (Table 2, Figure 3a). The specific details of another 10 plots in Sandu County could be seen in our previous study [21]. Each sample plot covered an area of 20 m by 20 m and trees with a diameter at breast height exceeding 5 cm were recorded. Additionally, four middle squares (5 m by 5 m) were established along the diagonal of each sample plot to examine the shrubs. Furthermore, four smaller plots (1 m by 1 m) were arranged in the center of each shrub layer sample to study the herbaceous plants [22].

## 3. Results

### 3.1. Diversity of Companion Plant Species in Three Different Types of Tea Gardens

In this study, a total of 24 companion plant species belonging to 16 families and 23 genera were found in ancient tea literature, including 12 species of tree plants, 7 species of shrub plants (with 5 species of them being both trees and shrubs), and 10 species of herbaceous plants (Table 3). 

The companion plants found in terrace tea gardens include 81 species belonging to 37 families and 74 genera, including 40 species of tree plants, 10 species of shrub plants (with 9 species of them exhibiting dual life forms), and 40 species of herbaceous plants (Table 3). According to the selected articles, intercropping economic trees (apples, mulberry trees, etc.) in tea terrace gardens demonstrated favorable strategic selection, while young terrace tea gardens (1–4 years old) were generally intercropped with leguminous plants (peanuts, peas, etc.).

The companion plants found in forest tea garden sample plots include 232 species belonging to 90 families and 178 genera, including 64 tree species, 98 shrub species, and 111 herbaceous species (Table 3, Table A1). In the sample plots of forest tea gardens we surveyed, the total vegetation coverage exceeded 92.44%. The JQ02 plot (Altitude 877 m) situated in Sandu County exhibited the highest species richness with 43 recorded species, while the CJ01 plot (Altitude 1301 m) located in Puding County presented the lowest species count of 25.

### 3.2. The Categories of Companion Plants

The companion plants could be divided into three categories. The first type was companion plants that have ecological value. For instance, tall trees such as *Firmiana simplex* (L.) W. Wight and *Morus alba* L. can provide shade for the tea plant, as tea plants prefer shade. Also, some companion plants could be multi-purpose (nitrogen fixing, fly attracting, water storage for tea, and so on). There was a multi-purpose tree written in several pieces of ancient literature named *Albizia chinensis* (Osh.) Merr, which not only can provide shade for tea but also attracts flies to avoid the gathering of flies around tea and contaminating the tea leaves. At the same time, it can also help regulate the soil moisture in the tea garden. The second type referred to aromatic plants, such as *Osmanthus* fragrans Lour. and *Yulania liliiflora* (Desr.) D. L. Fu. The third type included companion plants that have economic value (fruits and economic crops). In the terrace tea gardens, fruits trees like *Prunus salicina* L., *Mangifera indica* L., and *Punica granatum* L. were often used to provide shade for tea while economic crops like *Vicia faba* L., *Zea mays* L., and *Sorghum bicolor* (L.) Moench were often planted to change the microclimate of tea gardens and obtain economic benefits.

### 3.3. Dominant Family of Companion Plants in Three Different Types of Tea Gardens

**Figure 4:**
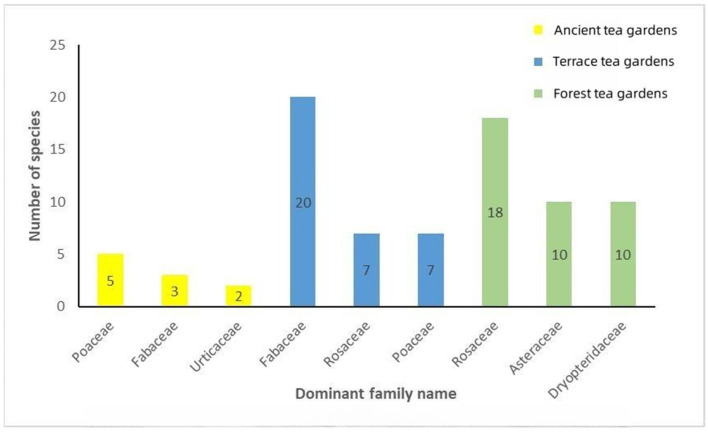
Families with many companion plant species appear in tea gardens.

According to ancient tea literature, a greater diversity of companion plant species was documented within the families of Poaceae, Urticaceae, and Fabaceae in ancient tea gardens. In terrace tea gardens, higher species diversity was observed in the families Fabaceae, Rosaceae, and Poaceae. Meanwhile, the predominant families were Rosaceae, Asteraceae, and Dryopteridaceae in sample plots of forest gardens in Guizhou (Figure 4). The findings demonstrated the prevalence of the Rosaceae family within both terrace and forest tea gardens, while the Poaceae and Fabaceae families exhibited dominance in ancient tea gardens and terrace tea gardens. 

### 3.4. The Common Family of Companion Plants in Three Different Types of Tea Gardens

The highest affinity in terms of companion plant species at the family taxonomic level was observed between ancient tea gardens and terrace tea gardens (Jaccard Index, JI = 0.23), and a high resemblance also occurred between terrace tea gardens and forest tea gardens (JI = 0.22), whereas the lowest similarity was detected across all three garden types (JI = 0.07). The level of similarity between ancient tea gardens and forest tea gardens fell within an intermediate range (JI = 0.12).

## 4. Discussion

Tea plants thrive beneath the canopy of arboreal vegetation in the forest and have evolved over an extended period of systematic cultivation to acquire distinctive genetic traits associated with shade endurance, temperature and humidity preferences, and the ability to efficiently harness diffused light [23]. Our research shows that the companion plants found in forest tea garden sample plots include 232 species belonging to 90 families and 178 genera, and there is a rich diversity of plant species in the Guizhou forest garden. It is found that as early as the Tang Dynasty in China, ancient people discovered that companion plants in tea gardens are beneficial to tea plants [24]. Appropriate plant diversity can improve the microclimate of tea gardens, improve the physical and chemical properties of soil, and increase beneficial insects in tea gardens [25,26,27,28], which further proves the importance of companion plants in tea gardens.

Currently, there are still serious issues that need to be addressed in the terrace tea garden. Primarily, commencing from the latter half of the 20th century, certain terrace tea gardens have employed substantial quantities of agrochemicals (pesticides, fertilizers) with the intention of achieving heightened tea yields. Regrettably, this practice has engendered soil microbiota, the dissipation of vital nutrients, and disruption to the ecological equilibrium of terrace tea gardens, thereby exerting a significant influence on the biodiversity of indigenous plant species [29,30]. In congruence with existing research findings, it has also come to our attention that a few forest tea gardens have suffered artificial degradation [31,32,33]. These gardens are susceptible to pests and diseases, resulting in suboptimal rates of tea plant resource utilization.

The intercropping of tea gardens has been practiced since ancient times and most of the intercropping plants in terrace tea gardens were fruits trees (pomegranate, loquat, and pear), economic trees (chestnut and rubber tree), legumes (broad bean, mung bean, and cowpea), and grain (corn and sorghum). In our research, we also found that only a small number of companion shrub plants were intercropped in terrace tea gardens. The reason for this may be that the tea planted in terrace tea gardens was mostly shrub tea plants, and there was a competitive relationship between shrubs. The intercropping of shrub species might lead to competition between them and tea plants for nutrient elements, which is unfavorable for tea growth and development [34]. Introducing intercropping practices encompassing clover, straw, and other herbaceous species offers a pragmatic method for diminishing soil erosion in terrace tea gardens, concurrently upholding soil element stability [35,36], and intercropping trees such as magnolia and chestnut in the middle of terrace tea gardens can contribute to the moderation of microclimatic conditions, fostering a cooler and more humid environment that benefits tea vegetation [37,38].

The Rosaceae family exhibits a relatively higher diversity of companion plant species within both terrace tea gardens and forest tea gardens. This phenomenon could be attributed to their considerable economic significance, expansive geographical distribution, and high endurance to climate change [39].

Comparing the three garden types, the family-level similarity is lowest among the companion herbaceous plants (which possess relatively greater species richness in forest tea gardens), while companion trees exhibit the highest level of similarity. This disparity could be attributed to the fact that certain companion herbs in forest tea gardens may be got rid of as weeds in terrace tea gardens, whereas certain companion trees play a favorable role in tea plant growth and garden development. The majority of the families observed in ancient tea gardens and terrace tea gardens can also be found in the forest tea gardens we investigated. These findings suggest that the inspiration for planting companion plant trees in ancient tea gardens and terrace tea gardens might have originated from natural forest tea gardens, which demonstrates the feasibility of promoting the vigorous development of forest tea gardens.

## 5. Conclusions

Our study indicates that the utilization of companion plants in tea gardens has been prevalent since antiquity, showcasing a diverse array of companion plant species. Notably, Poaceae, Fabaceae, and Rosaceae emerge as the most prevalent botanical families. These diverse companions can confer distinct advantages to tea cultivation. During the establishment of tea gardens, it is recommended to deliberate on approaches that ensure the preservation of the ecological balance and the optimal utilization of companion plant resources. This will facilitate the attainment of sustainable progress across a spectrum of tea garden contexts.

## Figures and Tables

**Table 1:** A summary of companion plants in ancient tea literature.

Serial Number	Ancient Tea Literatures Names	Author	The Year of Completion	Chinese Name of Companion Plants	Reference Fragment
1	Huayang Guo Zhi 华阳国志	Qu Chang	Eastern Jin Dynasty (348–354)	Mo Yu	There are fragrant konjac and fragrant tea in the garden. 园有芳蒻、香茗
2	Si Shi Zuan Yao 四时纂要	E Han	Late Tang and five Dynasties (Around 900)	Sang, Zhu, Su, Ji, Ma	Under the mulberry trees and bamboo shades, any place is suitable for planting… There is no restriction on planting ramie, millet, and barley. 桑下竹阴地种之皆可……面不妨种雄麻黍稷等
3	Twelve Rhymes of Tea Garden 茶园十二韵	Yucheng Wang	Song Dynasty (997)	Cong	Green onions share a garden. 青葱共一园
4	Tea Garden 茶园	Yuanchong Liu	Song Dynasty (1081)	Mei	The winter plum has not yet bloomed. 天寒梅未花
5	Elegy for Jun You Xu 徐君猷挽词	Shi Su	Song Dynasty (1083)	Liu, Zhu Zi	Alone, I came to plant willows after the snow. Through the bamboo grove, I walk once more to gather tea. 雪后独来栽柳处，竹间行复采茶时
6	Zi Liao 自料	Zi Zhang	Song Dynasty (1153–1221)	Sang	Planting tea should be done in a shaded area with mulberry trees 植茶要是依桑荫
7	Bei Yuan Bie Lu 北苑别录	Ruli Zhao	Song Dynasty (1196)	Tong	Tung wood remains, and the prosperity of tung wood is compatible with tea. 桐木则留桐木之兴与茶相宜
8	Jian Zao Yu Cha You Suo Zheng Zhi 监造御茶有所争执	Ji Xu	Song Dynasty (1162–1214)	Tong	The beautiful tung tree forest has tea trees under its shade. 修修桐树林，下荫茶树低
9	Journey at Noon 午行即事	Zengbo Li	Song Dynasty (1198–1265)	Mei, Song	A few white branches emerge from plum tree in the bamboo gate, and a few stacks of green trees lie horizontally on the pine outside the mountain. The people gradually pick tea on the hills of Longfu, while many households brew rice wine for sale at market. 竹门梅出数枝白,松外山横几叠青。人渐采茶登陇阜，家多酿秫市邮亭
10	Shi Xia Mountain Tea Blooms 石峡山茶盛开	Fengchen Fang	Song Dynasty (1221–1291)	Huo Shu	I am not alone while Phoenix Tree is under sunshine. 火树生阳我不孤
11	A Flower—Hangzhou Scenery 一枝花·杭州景	Hanqing Guan	Yuan Dynasty (1220–1300)	Dao	Tea Garden and Rice Paddy Path. 茶园稻陌
12	Duo Neng Bi Shi 多能鄙事	Ji Liu	Ming Dynasty (1311–1375)	Ma, Su Zhu Ma	When the tea plants are not yet matured, it can be planted with “Ma, Suand Zhu Ma” next to it 茶未成时其傍种麻、粟、苧诸不宿根布蔓之物皆可
13	Chun An Dao Zhong Yong Yu Lang Ba Yun 淳安道中用渔梁坝韵	Minzheng Cheng	Ming Dynasty (1446–1499)	Mai	The sparse plum trees stand on the bamboo bank, and the fine wheat embraces the tea garden 疏梅临竹岸，细麦拥茶园
14	Xi Hu Zhu Zhi Ci 西湖枝词	Zhideng Wang	Ming Dynasty (1535–1612)	Mei	Plant plum blossoms above and tea below 上种梅花下种茶
15	Cha Jie 茶解	Lin Luo	Ming Dynasty (1609)	Mei, Xin Yi, Gui, Yu Lan, Song, Zhu Zi	Such as osmanthus, plum, Xin Yi, magnolia, green pine, and emerald bamboo, and the like. 惟桂、梅、辛夷、玉兰、苍松、翠竹之类
16	Guang Dong Xin Yu 广东新语	Dajun Qu	Qing Dynasty (1630–1696)	Ku Ding Cha Shu	In the present day, people in the mountains often grow tea, occasionally complemented by Kudingcha tree. 今山中人率种茶,间以苦䔲
17	Yue Dong Wen Jian Lu 粤东闻见录	Qu Zhang	Qing Dynasty (1686–1740)	Ying Shu	In Xiqiao, most people grow tea, and in the tea Beds, there are Yingshu. 西樵多种茶，茶畦有蝇树
18	The Song of Planting Tea 种茶子歌	Tingdong Cao	Qing Dynasty (1699–1785)	Da Mai	Then he mixed it with barley. 下子继以大麦掺
19	Tea Picking Song 采茶曲	Bingkun Huang	Qing Dynasty (1832–1904)	Hu Ma	Drunk and fallen into seasame land, holding a pipa (a Chinese musical instrument). 醉倒胡麻抱琵琶
20	Zhong Cha Shuo 种茶说	Jingfan Zon	Qing Dynasty (1874)	Shu, Dou	When the tea tree is not yet lush, you can plant Shu and Dou next to it 茶树尚未茂盛之时，旁下空土犹可栽薯种豆
21	Fu Jun Nong Chan Kao Lue 抚郡农产考略	Gangde He	Qing Dynasty (1903)	Lan, Ju	Under the tea, fragrant orchids and graceful chrysanthemums can be planted, creating a clear and delightful aroma. 其(茶)下可植芳兰幽菊清芬之物

**Table 2:** Specific information of the sample plots in Puding County.

Region	Sample Plot Number	North Latitude	East Longitude	Altitude/m	Slope Aspect	Slope/°	Total Coverage/%
	DB	26°14′8.35″	105°34′24.58″	1287	SE	66	92
	CJ01	26°14′51.06″	105°34′28.47″	1301	E	69	95
	CJ02	26°14′48.33″	105°34′18.13″	1265	SW	65	96
Puding County	CJ03	26°14′50.17″	105°34′13.84″	1243	SW	69	98
	SZ01	26°13′53.68″	105°34′18.55″	1383	NE	77	93
	SZ02	26°14′26.45″	105°35′14.39″	1353	E	67	98
	SZ03	26°14′46.77″	105°35′15.74″	1370	SE	77	95
	XF	26°14′29.39″	105°34′17.11″	1307	N	76	92

**Table 3:** Companion plants in ancient gardens and terrace tea gardens.

Serial Number	Scientific Name	Family	Chinese Name	Life Form	Types of Tea Garden *
1	*Mangifera indica* L.	Anacardiaceae	Mang Guo	Tree	Terrace
2	*Alstonia scholaris* (L.) R. Br.	Apocynaceae	Tang Jiao Shu	Tree	Terrace
3	*Ilex Latifolia* Thunb.	Aquifoliaceae	Ku Deng	Tree	Ancient
4	*Pinellia ternate* (Thunb.) Breit.	Araceae	Ban Xia	Herbaceous	Terrace
5	*Areca catechu* L.	Arecaceae	Bing Lang	Tree	Terrace
6	*Hemerocallis citrina* Baroni	Asphodelaceae	Huang Hua Cai	Herbaceous	Terrace
7	Asteraceae sp.	Asteraceae	Ju	Herbaceous	Ancient, Forest
8	*Atractylodes macrocephala* Koidz.	Asteraceae	Bai Shu	Herbaceous	Terrace
9	*Artemisia argyi* Lévl. et Van.	Asteraceae	Ai Hao	Herbaceous	Terrace
10	*Alnus nepalensis* D.Don.	Betulaceae	Ni Bo Er Qi Mu	Tree	Terrace, Forest
11	*Raphanus sativus* L.	Brassicaceae	Luo Bu	Herbaceous	Terrace
12	*Brassica rapa* var. *glabra* Regel	Brassicaceae	Bai Cai	Herbaceous	Terrace
13	*Brassica napus* L.	Brassicaceae	You Cai	Herbaceous	Terrace
14	*Dioscorea alata* L.	Convolvulaceae	Shu	Herbaceous	Ancient, Terrace
15	*Cornus officinalis* Sieb. et Zucc	Cornaceae	Shan Zhu Yu	Tree, Shrub	Terrace
16	*Cunninghamia lanceolata* (Lamb.) Hook.	Cupressaceae	Shan Mu	Tree	Terrace
17	*Vatica mangachapoi* Blanco	Dipterocarpaceae	Qing Mei	Tree	Terrace
18	*Diospyros kaki* Thunb.	Ebenaceae	Shi Zi	Tree	Terrace
19	*Eucommia ulmoides* Oliv.	Eucommiaceae	Du Zhong	Tree	Terrace, Forest
20	*Hevea brasiliensis* (Willd. ex A. Juss.) Muell. Arg.	Euphorbiaceae	Xiang Jiao Shu	Tree	Terrace, Forest
21	*Ricinus communis* L.	Euphorbiaceae	Bi Ma	Shrub	Terrace
22	*Triadica sebifera* (L.) Small	Euphorbiaceae	Wu Bai	Tree, Shrub	Terrace
23	*Vernicia fordii* (Hemsl.) Airy Shaw	Euphorbiaceae	You Tong Shu	Tree	Terrace, Forest
24	*Albizia chinensis* (Osbeck) Merr.	Fabaceae	Ying Shu	Tree	Ancient
25	Fabaceae sp.	Fabaceae	Dou	Herbaceous	Ancient, Terrace
26	*Medicago sativa* L.	Fabaceae	Zi Hua Mu Xu	Herbaceous	Terrace
27	*Trifolium repens* L.	Fabaceae	Bai San Ye	Herbaceous	Terrace
28	*Astragalus sinicus* L.	Fabaceae	Zi Yun Ying	Herbaceous	Terrace
29	*Glycine max* (L.) Merr.	Fabaceae	Huang Dou	Herbaceous	Terrace
30	*Cassia rotundifolia* (Pers.) Greene	Fabaceae	Yuan Ye Jue Ming	Herbaceous	Terrace
31	*Indigofera spicata* Forssk.	Fabaceae	Pu Di Mu Lan	Herbaceous	Terrace
32	*Senna siamea* (Lam.) H. S. Irwin & Barneby	Fabaceae	Tie Dao Mu	Tree	Terrace
33	*Zenia insignis* Chun	Fabaceae	Ren Dou	Tree	Terrace
34	*Acacia richii* A. Gray	Fabaceae	Tai Wan Xiang Si Shu	Tree	Terrace
35	*Arachis hypogaea* L.	Fabaceae	Luo Hua Sheng	Herbaceous	Terrace
36	*Delonix regia* (Boj.) Raf.	Fabaceae	Huo Shu	Tree	Ancient
37	*Kummerowia striata* (Thunb.) Schindl.	Fabaceae	Ji Yan Cao	Herbaceous	Terrace
38	*Leucaena leucocephala* (Lam.) de Wit	Fabaceae	Yin He Huan	Tree	Terrace
39	*Macroptilium lathyroides* (L.) Urban	Fabaceae	Da Yi Dou	Herbaceous	Terrace
40	*Pisum sativum* L.	Fabaceae	Wan Dou	Herbaceous	Terrace
41	*Phaseolus calaratus* Roxb.	Fabaceae	Zhu Dou	Herbaceous	Terrace
42	*Vicia faba* L.	Fabaceae	Can Dou	Herbaceous	Terrace
43	*Vicia sativa* L.	Fabaceae	Ye Wan Dou	Herbaceous	Terrace
44	*Vigna radiata* L. Wilczek	Fabaceae	Lv Dou	Herbaceous	Terrace
45	*Vigna unguiculata* (L.) Walp.	Fabaceae	Gang Dou	Herbaceous	Terrace
46	*Castanea mollissima* Blume	Fagaceae	Ban Li	Tree	Terrace, Forest
47	*Ginkgo biloba* L.	Ginkgoaceae	Yin Xing	Tree	Terrace, Forest
48	*Cinnamomum camphora* (L.) Presl	Lauraceae	Zhang Shu	Tree	Terrace, Forest
49	*Litsea cubeba* (Lour.) Pers.	Lauraceae	Shan Ji Jiao	Tree, Shrub	Terrace
50	*Ocimum basilicum* L.	Labiatae	Luo Le	Herbaceous	Terrace
51	*Perilla frutescens* (L.) Britt.	Labiatae	Zi Su	Herbaceous	Terrace
52	*Agastache rugosa* (Fisch. & C. A. Mey.) Kuntze	Labiatae	Huo Xiang	Herbaceous	Terrace
53	*Salvia japonica* Thunb.	Labiatae	Shu Wei Cao	Herbaceous	Terrace
54	*Allium sativum* L.	Liliaceae	Da Suan	Herbaceous	Terrace
55	*Alliun fistulosum* L.	Liliaceae	Cong	Herbaceous	Ancient
56	*Punica granatum* L.	Lythraceae	Shi Liu	Tree, Shrub	Terrace
57	*Firmiana simplex* (L.) W. Wight	Malvaceae	Wu Tong	Tree	Ancient
58	*Yulania denudate* (Desr.) D. L. Fu	Magnoliaceae	Yu Lan	Tree	Ancient, Forest
59	*Yulania liliiflora* (Desr.) D. L. Fu	Magnoliaceae	Xin Yi	Shrub	Ancient
60	*Toona sinensis* (A. Juss.) Roem	Meliaceae	Xiang Chun	Tree	Terrace
61	*Morus alba* L.	Moraceae	Sang	Tree, Shrub	Ancient, Terrace, Forest
62	*Musa acuminata* Colla	Musaceae	Xiang Jiao	Herbaceous	Terrace
63	*Morella rubra* Lour.	Myricaceae	Yang Mei	Tree	Terrace, Forest
64	*Osmanthus fragrans* Lour.	Oleacea	Gui	Tree, Shrub	Ancient, Terrace
65	Orchidaceae sp.	Orchidaceae	Lan	Herbaceous	Ancient, Terrace
66	*Sesamum indicum* L.	Pedaliaceae	Hu Ma	Shrub	Ancient
67	*Pinus elliottii* Engelm.	Pinaceae	Shi Di Song	Tree	Terrace
68	*Pinus* sp.	Pinaceae	Song	Tree	Ancient, Terrace, Forest
69	*Pinus taeda* L.	Pinaceae	Hou Ju Song	Tree	Terrace
70	*Bambusoideae* sp.	Poaceae	Zhu Zi	Herbaceous	Ancient, Forest
71	*Hordeum vulgare* L.	Poaceae	Da Mai	Herbaceous	Ancient
72	*Oryza sativa* L.	Poaceae	Dao	Herbaceous	Ancient, Terrace
73	*Saccharum officinarum* L.	Poaceae	Gan Zhe	Herbaceous	Terrace
74	*Lolium multiflorum* Lamk.	Poaceae	Duo Hua Hei Mai Cao	Herbaceous	Terrace
75	*Paspalum notatum* Flugge	Poaceae	Bai Xi Cao	Herbaceous	Terrace
76	*Panicum miliaceum* L.	Poaceae	Ji	Herbaceous	Ancient
77	*Setaria italica* var. *germanica* (Mill.) Schred.	Poaceae	Su	Herbaceous	Ancient
78	*Sorghum bicolor* (L.) Moench	Poaceae	Gao Liang	Herbaceous	Terrace
79	*Zea mays* L.	Poaceae	Yu Mi	Herbaceous	Terrace
80	*Secale cereale* L.	Poaceae	Hei Mai	Herbaceous	Terrace
81	*Eriobotrya japonica* (Thunb.) Lindl.	Rosaceae	Pi Pa	Tree	Terrace, Forest
82	*Malus pumila* Mill.	Rosaceae	Ping Guo	Tree	Terrace
83	*Prunus mume* Siebold & Zucc.	Rosaceae	Mei	Tree, Shrub	Ancient, Terrace, Forest
84	*Prunus salicina* L.	Rosaceae	Li	Tree, Shrub	Terrace, Forest
85	*Crataegus pinnatifida* Bunge	Rosaceae	Shan Zha	Tree	Terrace
86	*Prunus* sp.	Rosaceae	Ying Tao	Tree	Terrace
87	*Pyrus bretschneideri* Rehd.	Rosaceae	Li Shu	Tree	Terrace
88	*Citrus reticulata* Blanco	Rutaceae	Gan Ju	Tree, Shrub	Terrace, Forest
89	*Populus* sp.	Salicaceae	Yang Shu	Tree	Terrace, Forest
90	*Salix* sp.	Salicaceae	Liu	Tree	Ancient
91	*Dimocarpus longan* L.	Sapindaceae	Long Yan	Tree	Terrace
92	*Lucuma nervosa* A.DC.	Sapotaceae	Dan Huang Guo	Tree	Terrace
93	*Paulownia fortunei* (Seem.) Hemsl	Scrophulariaceae	Bai Hua Pao Tong	Tree	Terrace, Forest
94	*Capsicum annuum* L.	Solanaceae	La Jiao	Herbaceous	Terrace
95	*Laportea* sp.	Urticaceae	Ai Ma	Tree, Shrub	Ancient
96	*Boehmeria* sp.	Urticaceae	Zhu Ma	Tree, Shrub	Ancient
97	*Vitis vinifera* L.	Vitaceae	Pu Tao	Herbaceous	Terrace, Forest

* Note: If the companion plants presented here also existed in forest gardens, then it was also marked with “forest” in the last column.

## Data Availability

Data is contained within the article and Appendix A.

## Appendix A

**Table A1:** Companion plants in forest tea gardens in Puding County, Guizhou Province, China.

Serial Number	Scientific Name	Family	Chinese Name	Life Form
1	*Alternanthera philoxeroides* (Mart.) Griseb	Amaranthaceae	Kon Xin Lian Zi Cao	Herbaceous
2	*Toxicodendron vernicifluum* (Stokes) F. A. Barkl.	Anacardiaceae	Qi Shu	Tree
3	*Aralia echinocaulis* Hand. Mazz.	Araliaceae	Chi Jin Song Mu	Tree
4	*Artemisia carvifolia* Buch.-Ham. ex Roxb.	Asteraceae	Qing Hao	Herbaceous
5	*Artemisia lavandulifolia* DC.	Asteraceae	Ye Ai Hao	Herbaceous
6	*Conyza sumatrensis* (Retz.) Walker	Asteraceae	Su Men Bai Jiu Cao	Herbaceous
7	*Erigeron acer* L.	Asteraceae	Fei Peng	Herbaceous
8	*Eupatorium coelestinum* L.	Asteraceae	Po Hua Cao	Herbaceous
9	*Gnaphalium hypoleucum* DC.	Asteraceae	Qiu Shu Qu Cao	Herbaceous
10	*Senecio scandens* Buch.-Ham. ex D. Don	Asteraceae	Qian Li Guang	Herbaceous
11	*Blumea balsamifera* (L.) DC.	Asteraceae	Ai Na Cao	Herbaceous
12	*Sphaeranthus africanus* L.	Asteraceae	Dai Xinh Cao	Herbaceous
13	*Athyrium otophorum* (Miq.) Koidz	Athyriaceae	Guang Ti Gai Jue	Herbaceous
14	*Betula luminifera* H. Winkl.	Betulaceae	Guang Ye Hua	Tree
15	*Catalpa bungei* C. A. Mey.	Bignoniaceae	Qiu	Tree
16	*Pachysandra axillaris* Franch.	Buxaceae	Ban Deng Guo	Shurb
17	*Lonicera fulvotomentosa* Hsu et S. C. Cheng	Caprifoliaceae	Huang He Mao Ren Dong	Shurb
18	*Lonicera hypoglauca* Miq.	Caprifoliaceae	Gu Xian Ren Dong	Shurb
19	*Viburnum fordiae* Hance	Caprifoliaceae	Nan Fang Jia Mi	Shurb
20	*Cerastium fontanum* Baumg.	Caryophyllaceae	Con Sheng Juan Er	Herbaceous
21	*Stellaria vestita* Kurz.	Caryophyllaceae	Jing Gu Cao	Herbaceous
22	*Cornus macrophylla* (Wall.) Sojak	Cornaceae	Lai Mu	Tree
23	*Capsella bursa-pastoris* (L.) Medic.	Cruciferae	Ji Cai	Herbaceous
24	*Dioscorea bulbifera* L.	Dioscoreaceae	Huang Du	Herbaceous
25	*Cyrtomium caryotideum* (Wall.) Presl	Dryopteridaceae	Chi Ci Guan Zhong	Herbaceous
26	*Cyrtomium fortunei* J. Sm.	Dryopteridaceae	Guan Zhong	Herbaceous
27	*Dryopteris championii* (Benth.) C. Chr.	Dryopteridaceae	Kua Ling Ling Mao Jue	Herbaceous
28	*Dryopteris setosa* (Thunb.) Akasawa	Dryopteridaceae	Liang Se Ling Mao Jue	Herbaceous
29	*Polystichum xiphophyllum* (Baker) Diels	Dryopteridaceae	Jian Ye Er Jue	Herbaceous
30	*Elaeagnus pungens* Thunb.	Elaeagnaceae	Hu Tui Zi	Herbaceous
31	*Hylodesmum podocarpum* (Candolle) H. Ohashi & R. R. Mill	Fabaceae	Chang Bin Shan ma Huang	Shurb
32	*Castanopsis chinensis* (Sprengel) Hance	Fagaceae	Zhui	Shurb
33	*Clinopodium chinense* (Benth.) O. Ktze.	Labiatae	Feng Lun Cai	Herbaceous
34	*Clinopodium gracile* (Benth.) O. Ktze.	Labiatae	Xi Feng Lun Cai	Herbaceous
35	*Stauntonia* sp.	Lardizabalaceae	Niu Teng	Herbaceous
36	*Cinnamomum bodinieri* H. Lév.	Lauraceae	Hou Zhang	Shurb
37	*Phoebe zhennan* S. K. Lee & F. N. Wei	Lauraceae	Nan Mu	Shurb
38	*Ophiopogon japonicus* (L. f.) Ker-Gawl.	Liliaceae	Mai Dong	Herbaceous
39	*Stenoloma chusanum* Ching	Lindsaeaceae	Wu Jue	Herbaceous
40	*Palhinhaea cernua* (L.) Vasc. & Franco	Lycopodiaceae	Chui Sui Shi Song	Herbaceous
41	*Toona sinensis* (A. Juss.) Roem.	Meliaceae	Xiang Chun	Shurb
42	Broussonetia papyrifera (L.) Vent	Moraceae	Hou Shu	Herbaceous
43	*Ficus heteromorpha* Hemsl.	Moraceae	Yi Ye Rong	Shurb
44	*Humulus scandens* (Lour.) Merr.	Moraceae	Lv Cao	Herbaceous
45	*Morus australis* Poir.	Moraceae	Ji Sang	Shurb
46	*Camptotheca acuminata* Decne.	Nyssaceae	Xi Shu	Shurb
47	*Osmunda japonica* Thunb.	Osmundaceae	Zhi Qi	Herbaceous
48	*Oxalis corniculata* L.	Oxalidaceae	Chu Jiang Cao	Herbaceous
49	*Plantago asiatica* L.	Plantaginaceae	Che Qian Cao	Herbaceous
50	*Arthraxon hispidus* (Trin.) Makino	Poaceae	Jing Cao	Herbaceous
51	*Eremochloa ciliaris* (L.) Merr.	Poaceae	Wu Gong Cao	Herbaceous
52	*Heteropogon contortus* (L.) P. Beauv. ex Roem. et Schult.	Poaceae	Huang Mao	Herbaceous
53	*Microstegium nodosum* (Kom.) Tzvel.	Poaceae	Xiu Zhu	Herbaceous
54	*Miscanthus sinensis* Anderss.	Poaceae	Mang	Herbaceous
55	*Fagopyrum dibotrys* (D.Don)Hara	Polygonaceae	Jin Qiao Mai	Herbaceous
56	*Fallopia multiflora* (Thunb.) Harald.	Polygonaceae	He Shou Wu	Herbaceous
57	*Pteris cretica* L.	Pteridaceae	Feng Wei Jue	Herbaceous
58	*Pteridium aquilinum* (L.) Kuhn	Pteridiaceae	Jue	Herbaceous
59	*Ranunculus sieboldii* Miq.	Ranunculaceae	Yang Zi Mao Geng	Herbaceous
60	*Agrimonia pilosa* Ldb.	Rosaceae	Long Ya Cao	Herbaceous
61	*Duchesnea indica* (Andr.) Focke	Rosaceae	Shei Mei	Herbaceous
62	*Pyrus pashia* F. Schmidt	Rosaceae	Ci Li	Shurb
63	*Cerasus pseudocerasus* (Lindl.) G. Don	Rosaceae	Ying Tao	Tree, Shurb
64	*Rosa cymosa* Tratt.	Rosaceae	Xiao Guo Qiang Wei	Shurb
65	*Rubus multiflora* Thunb.	Rosaceae	Ye Qiang Wei	Shurb
66	*Rubus coreanus* Miq.	Rosaceae	Cha Tian Pao	Shurb
67	*Rubus ellipticus* var. *obcordatus* (Franch.) Focke	Rosaceae	Zai Yang Pao	Shurb
68	*Rubus pectinellus* Maxim.	Rosaceae	Huang Pao	Shurb
69	*Rubus swinhoei* Hance	Rosaceae	Mu Mei	Shurb
70	*Rubia cordifolia* L.	Rubiaceae	Qian Cao	Herbaceous
71	*Evodia fargesii* Dode	Rutaceae	Shu Ye Wu Yu	Shurb
72	*Populus adenopoda* Maxim.	Salicaceae	Xiang Ye Yang	Tree, Shurb
73	*Populus tremula* var. *villosa* Carr.	Salicaceae	Bai Yang	Tree, Shurbs
74	*Koelreuteria paniculata* L.	Sapindaceae	Luan Shu	Tree
75	*Houttuynia cordata* Thunb.	Saururaceae	Zhe Er Geng	Herbaceous
76	*Selaginella tamariscina* (P. Beauv.) Spring	Selaginellaceae	Juan Bai	Herbaceous
77	*Onychium japonicum* (Thunb.) Kunze	Sinopteridaceae	Ye Zi Jin Wei Fen	Herbaceous
78	*Solanum virginianum* L.	Solanaceae	Huang Guo Qie	Shurb
79	*Cryptomeria fortunei* Miquel	Taxodiaceae	Liu Shan	Tree, Shurb
80	*Cunninghamia lanceolata* (Lamb.) Hook.	Taxodiaceae	Shan Mu	Tree, Shurb
81	*Cyclosorus acuminatus* (Houtt.) Nakai	Thelypteridaceae	Jing Jian Mao Jue	Herbaceous
82	*Phegopteriis decursive-pinnata* (Ven Hall) Fée	Thelypteridaceae	Yan Yu Luan Guo Jue	Herbaceous
83	*Cryptotaenia japonica* Hassk.	Umbelliferae	Ya Er Qing	Herbaceous
84	*Debregeasia orientalis* C. J. Chen	Urticaceae	Shui Ma	Shurb
85	*Gonostegia hirta* (Bl.) Miq.	Urticaceae	Luo Mi Tuan	Herbaceous
86	*Pilea notata* C. H. Wright	Urticaceae	Len Shui Hua	Shurb
87	*Urtica fissa* E. Pritz.	Urticaceae	Qian Ma	Shurb
88	*Viola philippica* Cav.	Violaceae	Zhi Hua Di Ding	Herbaceous
89	*Cayratia japonica* (Thunb.) Raf.	Vitaceae	Wu Lian Mei	Herbaceous

Note: If the companion plants in forest gardens were already listed in Table 3 (marked with “forest”), then no duplication was presented in Appendix A (Table A1).
